# Rush Medical College

**Published:** 1886-04

**Authors:** 


### LOCAL NEWS.

RUSH MEDICAL COLLEGE.—The Annual Commencement of Rush Medical College was held on Tuesday, February 16. Diplomas were conferred on 156 graduates. Five diplomas were conferred *pro causa honoris*. The total number of matriculates during the winter session was 404; during the preceding spring term, 138.

Professor Norman Bridge, M.D., formerly Professor of Hygiene, and Adjunct Professor of the Principles and Practice of Medicine, has been elected Professor of General Pathology.

Hereafter, practical work in the physiological and pathological laboratories will be required of undergraduates.

